# An Antibiotic-Responsive Mouse Model of Fulminant Ulcerative Colitis 

**DOI:** 10.1371/journal.pmed.0050041

**Published:** 2008-03-04

**Authors:** Silvia S Kang, Seth M Bloom, Lyse A Norian, Michael J Geske, Richard A Flavell, Thaddeus S Stappenbeck, Paul M Allen

**Affiliations:** 1 Department of Pathology and Immunology, Washington University School of Medicine, St. Louis, Missouri, United States of America; 2 Section of Immunobiology, Yale University School of Medicine, New Haven, Connecticut, United States of America; 3 Howard Hughes Medical Institute, Yale University School of Medicine, New Haven, Connecticut, United States of America; University of Oslo, Norway

## Abstract

**Background:**

The constellation of human inflammatory bowel disease (IBD) includes ulcerative colitis and Crohn's disease, which both display a wide spectrum in the severity of pathology. One theory is that multiple genetic hits to the host immune system may contribute to the susceptibility and severity of IBD. However, experimental proof of this concept is still lacking. Several genetic mouse models that each recapitulate some aspects of human IBD have utilized a single gene defect to induce colitis. However, none have produced pathology clearly distinguishable as either ulcerative colitis or Crohn's disease, in part because none of them reproduce the most severe forms of disease that are observed in human patients. This lack of severe IBD models has posed a challenge for research into pathogenic mechanisms and development of new treatments. We hypothesized that multiple genetic hits to the regulatory machinery that normally inhibits immune activation in the intestine would generate more severe, reproducible pathology that would mimic either ulcerative colitis or Crohn's disease.

**Methods and Findings:**

We generated a novel mouse line (dnKO) that possessed defects in both TGFβRII and IL-10R2 signaling. These mice rapidly and reproducibly developed a disease resembling fulminant human ulcerative colitis that was quite distinct from the much longer and more variable course of pathology observed previously in mice possessing only single defects. Pathogenesis was driven by uncontrolled production of proinflammatory cytokines resulting in large part from T cell activation. The disease process could be significantly ameliorated by administration of antibodies against IFNγ and TNFα and was completely inhibited by a combination of broad-spectrum antibiotics.

**Conclusions:**

Here, we develop to our knowledge the first mouse model of fulminant ulcerative colitis by combining multiple genetic hits in immune regulation and demonstrate that the resulting disease is sensitive to both anticytokine therapy and broad-spectrum antibiotics. These findings indicated the IL-10 and TGFβ pathways synergize to inhibit microbially induced production of proinflammatory cytokines, including IFNγ and TNFα, which are known to play a role in the pathogenesis of human ulcerative colitis. Our findings also provide evidence that broad-spectrum antibiotics may have an application in the treatment of patients with ulcerative colitis. This model system will be useful in the future to explore the microbial factors that induce immune activation and characterize how these interactions produce disease.

## Abstract 2

Paul Allen and colleagues describe the development of a mouse model of fulminant ulcerative colitis with multiple genetic hits in immune regulation which can be moderated by anti-cytokine therapy and broad-spectrum antibiotics.

## Editors' Summary

**Background.:**

Inflammatory bowel disease (IBD), a group of disorders characterized by inflammation (swelling) of the digestive tract (the tube that runs from the mouth to the anus), affects about 1.4 million people in the US. There are two main types of IBD. In Crohn's disease, which can affect any area of the digestive tract but most commonly involves the lower part of the small intestine (small bowel), all the layers of the intestine become inflamed. In ulcerative colitis, which primarily affects the colon (large bowel) and the rectum (the part of the bowel closest to the anus), only the lining of the bowel becomes inflamed, the cells in this lining die, and sores or ulcers form. Both types of IBD most commonly develop between the ages of 15 and 35 years, often run in families, and carry an increased risk of cancer. Symptoms—usually diarrhea and abdominal cramps—can be mild or severe and the disorder can develop slowly or suddenly. There is no medical cure for IBD, but drugs that modulate the immune system (for example, corticosteroids) can help some people. Some people benefit from treatment with drugs that specifically inhibit “proinflammatory cytokines,” proteins made by the immune system that stimulate inflammation (for example, TNFα and INFγ). When medical therapy fails, surgery to remove the affected part of the bowel may be necessary.

**Why Was This Study Done?:**

Exactly what causes IBD is not clear, but people with IBD seem to have an overactive immune system. The immune system normally protects the body from harmful substances but in IBD it mistakenly recognizes the food substances and “good” bacteria that are normally present in the human gut as foreign and hence reacts against them. As a result, immune system cells accumulate in the lining of the bowel and cause inflammation. Several different pathways usually prevent inappropriate immune activation, so could IBD be caused by alterations in one or several of these immune regulatory pathways? In previous studies, mice with a defect in just one pathway have developed mild intestinal abnormalities but not the problems seen in the most severe forms of IBD. In this study, therefore, the researchers have generated and characterized a new mouse line with defects in two immune regulatory pathways to see whether this might be a better animal model of human IBD.

**What Did the Researchers Do and Find?:**

To make their new mouse line, the researchers mated mice that had a defective TGFβ signaling pathway in their T lymphocytes with mice that had a defective IL-10 signaling pathway. Both these pathways are anti-inflammatory, and mice with defects in either pathway develop mild and variable inflammation of the colon (colitis) by age 3–4 months. By contrast, the doubly defective mice (dnKO mice) failed to thrive, lost weight, and died by 4–6 weeks of age. The colons of 4- to 5-week old dnKO mice were inflamed and ulcerated (some changes were visible in 3-week-old mice) and contained many immune system cells. Mice with a single defective signaling pathway had no gut abnormalities at this age. The dnKO mice, just like people with IBD, had higher than normal blood levels of IFNγ, TNFα, and other proinflammatory cytokines; these raised levels were the result of abnormal lymphocyte activation. Treatment of the dnKO mice with a combination of agents that neutralize IFNγ and TNFα (anti-cytokine therapy) greatly reduced the colitis seen in these mice; neutralization of IFNγ alone had some beneficial effects, but neutralization of TNFα alone had no effect. Finally, early treatment of the dnKO mice with broad-spectrum antibiotics completely inhibited colitis.

**What Do These Findings Mean?:**

These findings suggest that dnKO mice are a good model for fulminant (severe and rapidly progressing) ulcerative colitis and support the idea that IBD involves multiple genetic defects in immune regulation. They also indicate that the IL-10 and the TGFβ signaling pathways normally cooperate to inhibit the inappropriate immune responses to intestinal bacteria seen in IBD. This new mouse model should help researchers unravel what goes wrong in IBD and should also help them develop new treatments for ulcerative colitis. More immediately, these findings suggest that combined anti-cytokine therapy may be a better treatment for ulcerative colitis than single therapy. In addition, they suggest that clinical studies should be started to test whether broad-spectrum antibiotics can ameliorate ulcerative colitis in people.

**Additional Information.:**

Please access these Web sites via the online version of this summary at http://dx.doi.org/10.1371/journal.pmed.0050041.

- The Medline Plus Encyclopedia has pages on Crohn's disease and on ulcerative colitis (in English and Spanish)
- Information is available from the UK National Health Service Direct Health Encyclopedia about Crohn's disease and ulcerative colitis
- The US National Institute of Diabetes and Digestive and Kidney Diseases provides information on Crohn's disease and ulcerative colitis
- Information and support for patients with inflammatory bowel disease and their caregivers is provided by the Crohn's and Colitis Foundation of America and by the UK National Association for Colitis and Crohn's Disease

## Introduction

Inflammatory bowel disease (IBD) is an idiopathic disease of the intestinal tract with the hallmark features of mucosal inflammation and loss of barrier function. Although the exact mechanisms for IBD induction and progression are not completely understood, unchecked immune responses to enteric bacteria are critical for pathogenesis (reviewed in [1]). The clinical presentation of IBD occurs over a wide spectrum from chronic indolent to acute fulminant disease. A recent hypothesis is that susceptibility to disease, including the variation in presentation, may be a result of alterations of multiple pathways that control intestinal mucosal homeostasis [2].

At least ten different murine models of IBD involving single gene manipulations have been developed and demonstrate the role for proper development/maintenance of specific subsets of T cells and control of inflammatory cytokine production in regulation of disease (reviewed in [1,2]), as well as revealing a crucial role for enteric bacteria in inducing disease (e.g., [3–6]). While a variety of existing models recapitulate aspects of human IBD, many are limited by variable penetrance, delayed development of characteristic disease pathology, and, in some cases, induction of nonspecific severe multiorgan autoimmunity [3–5,7–9]. Additionally, none of these models consistently and predictably mimics the most severe and acute presentation of either ulcerative colitis or Crohn's disease. This animal-to-animal variability in disease penetrance and severity poses a significant challenge for efforts to experimentally manipulate the course of disease in these models.

Because the current number of known genetic manipulations that can produce a partial IBD phenotype is at least ten, and the minimum number of multiple genetic hits is two, the resulting number of possible two-hit combinations is 45. We chose to utilize a model involving the loss of the IL-10 signaling pathway because uncharacterized loci that increased the severity of colitis in IL-10^−/−^ mice have been defined using a genetic approach [10,11]. These findings support the hypothesis that alterations of multiple loci can exacerbate pathology both in mouse models and human IBD. Because both IL-10 and TGFβ signaling pathways have been demonstrated to be anti-inflammatory, in part through control of proinflammatory cytokines such as IFNγ and TNFα [12–16], we hypothesized that genetic ablation of these two pathways would synergize to elicit more acute pathology than loss of either pathway alone.

## Materials and Methods

### Mice

All female or male C57BL/6 (WT) (The Jackson Laboratory), dominant negative TGFβRII mice (supplied by RAF) [8], and CRF2–4-deficient (IL-10R2^−/−^) mice (Genentech) [9] were on the C57BL/6 background. Importantly, all mice used in these experiments were bred and housed in a specific pathogen-free barrier facility at Washington University. Animal protocols were reviewed and approved by the Washington University animal studies committee. Animals were used between 3–6 wk of age for this study, with the exception of long-term measurements that were taken out to approximately 3–3.5 mo of age. The CRF2–4^−/−^ mice lack the IL-10R2 receptor protein and are referred to as IL-10R2^−/−^ in this report. The dominant negative TGFβRII mice (referred to as dnTGFβRII), express a dominant negative TGFβRII solely in the CD4 and CD8 compartment. Mice that were unresponsive to IL-10R2 signaling in all compartments and TGFβ signaling specifically in the T cell compartment were generated by breeding dnTGFβRII mice with IL-10R2^−/−^ to yield a novel strain of mouse, dnTGFβRII × IL-10R2^−/−^, referred to as dnKO mice. The breeding scheme involved mating dnTGFβRII × IL-10R2^+/−^ mice with IL-10R2^−/−^ mice to generate four genotypes of littermates: dnKO, IL-10R2^−/−^, dnTGFβRII × IL-10R2^+/−^ (referred to as dnTGFβRII), and IL-10R2^+/−^ (referred to as WT) mice.

### Weight Loss Measurements

Mice were weighed every day or every other day using a portable electronic Ohaus scale (VWR International). Weights were recorded to a tenth of a gram.

### Harvesting and Preparation of Intestines for Hematoxlyin and Eosin Staining

Mice were humanely killed and large intestines were harvested, flushed with PBS, and fixed with Bouin's fixative (70% picric acid/25% formaldehyde [37%]/5% glacial acetic acid). Intestines were cut from cecum to rectum, splayed out, and pinned with insect pins (Carolina Biological) onto a square Petri dish filled with wax (Carolina Biological). Intestines were incubated in Bouin's fix for 4–8 h and then placed in 70% ethanol overnight. Pictures of the whole view or whole mounts of the intestines were taken using either a digital camera or an Olympus 52 × 12 whole mount camera (Diagnostic Instruments), respectively. Intestines were paraffin embedded, cut in 5-μm sections, and stained with hematoxylin and eosin (HE) at the histology core (Washington University, St. Louis, Missouri, United States).

### Gross Morphologic and Microscopic Parameters for Scoring the Severity of Mucosal Damage

Images of the rectal mucosal surface were obtained using a dissecting stereoscope and were scored in a blinded fashion by an anatomic pathologist (TSS) using the following scoring system: 0, normal; 1, focal ulcers present; 2, ulcers and diffuse, mild mucosal thickening; and 3, ulcers and diffuse, severe mucosal thickening. Blinded microscopic analysis of histologic HE-stained sections was performed using an Olympus B×51 microscope at 200× magnification to determine rectal crypt heights, crypt widths, and goblet cell number and 400× to determine surface epithelial heights and the number of mitotic and apoptotic bodies. Only crypt-surface units that were well oriented (i.e., the entire length of the crypt could be discerned) were evaluated.

### Isolation of Cells from the Lamina Propria/Mucosally Associated Lymphoid Tissue

Intestines were harvested and flushed with PBS. The cecum, descending colon, and rectum were cut into small pieces and washed six times with CMF-HEPES solution (0.015 M HEPES/Ca^2+^ and Mg^2+^ free HBSS [pH 7.2]). Intestines were incubated with CMF/BGS/EDTA (10% bovine growth serum/0.015 M HEPES/5 mM EDTA/Ca^2+^ and Mg^2+^ free HBSS [pH 7.2]) solution at 37 °C for 15 min, shaking at ∼275 rpm. After removing the supernatants, the process was repeated three more times with CMF/BGS/EDTA. The remaining intestinal tissue pieces were shaken for 5 min at 37 °C in RPMI/10% BGS. After removal of the media, the tissue was incubated with RPMI/10% BGS containing 1 mg/ml collagenase type IV (Sigma) for 1 h at 37 °C, shaking at ∼275 rpm. This process was repeated two more times and supernatants containing the lamina propria/mucosally associated lymphoid tissue (MALT) cells were collected, centrifuged, and resuspended in CMF-HEPES at each step. For cytokine analysis, a 40%–100% Percoll gradient was also run to remove any dead cells or debris.

### Flow Cytometric Analysis

Cells were surface stained at 4 °C for 10–15 min with antibodies against CD4 FITC/PE or /PE-Cy5, CD8 FITC/PE or /PECy5, Gr1 FITC, CD25PE, CD45.2 FITC or PE, CD62L PE, B220 FITC, and DX5 biotin-conjugated antibodies (BD Pharmingen or Biolegend). For biotinylated antibodies, streptavidin PE or streptavidin APC conjugated secondaries (Caltag) were added to the cells in a second incubation step. Cells were analyzed by flow cytometry on a FACScan or a FACsCaliber (BD Biosciences) using CellQuest analysis software. For studies of live cells, 7AAD (Sigma) was added prior to analysis.

### Intracellular Cytokine Staining

Cells were harvested, centrifuged, and then resuspended in RPMI 1640 supplemented with 10% FCS (HyClone), 2 mM Glutamax (Life Technologies), 0.5 μM 2-ME (Sigma), and 50 μg/ml gentamicin (Invitrogen Life Technologies) containing 50 ng/ml PMA and 500 ng/ml ionomycin (Sigma) and incubated for 4 h at 37 °C. Brefeldin A (Sigma) was added during the last 2 h of culture at 10 μg/ml. Cells were surface stained for CD4 or CD8 for 15 min at 4 °C (BD Pharmingen). Stained cells were fixed in 2%–4% paraformaldehyde for 20 min at RT prior to permeabilization with 0.5% saponin/1% BSA/PBS. Intracellular staining for the cells was conducted at RT for 30 min using anti-IL-17 PE (4 μg/ml) (Biolegend), anti-IFNγ FITC (2.5 μg/ml), and anti-TNFα PE (4 μg/ml), conjugated antibodies.

### Serum Cytokine Concentrations

Blood from mice was clotted on ice for 1 h. The samples were then centrifuged at 10,000 rpm for 5 min, and the serum was removed. All serum samples were stored at 20 °C prior to analysis. The concentrations of IFNγ, TNFα, and IL-6 was determined using an inflammatory cytokine cytometric bead analysis (CBA) kit (BD Pharmingen) and analyzed on a FACScan (BD biosciences) using CBA analysis software. The lower limit of detection for all cytokines was set to 20 pg/ml, the minimum quantifiable value, as described in the kit manual.

### CD4^+^ T Cell Transfer

CD4^+^ T cells were purified from the lymph nodes of 3-wk-old WT B6, IL-10R2^−/−^, dnTGFβRII, and dnKO mice by positive selection using Miltenyi CD4 beads according to the manufacturer's protocol. 2 × 10^6^ purified CD4^+^ T cells were transferred into B6.RAG1^−/−^ mice by IP injection, and the weights of the mice were determined every other day. Five weeks post-T cell transfer, mice were humanely killed and the intestines were harvested, flushed with PBS, fixed with Bouin's fixative, and prepared according to the methods discussed above.

### Cytokine Neutralization

dnKO mice were injected IP with either 1 mg of a hamster anti-PIP isotype control, hamster anti-IFNγ (H22) [17], hamster anti-TNFα (TN3–19.12) [18], or a combination of anti-IFNγ and anti-TNFα at 2 wk and 3 wk of age. All neutralizing antibodies were a generous gift from Robert Schreiber. Intestines were harvested from mice at 4 wk of age, fixed in Bouin's solution, paraffin embedded, and HE stained.

### Antibiotic Experiments

Mice received drinking water containing 0.66 mg/ml ciprofloxacin [19] and 2.5 mg/ml metronidazole (Sigma) [20] beginning at 24 d of age. Previous reports (e.g., [21]) have suggested occasional refusal of mice to drink water that contains antibiotics, so we included 20 mg/ml sugar-sweetened grape Kool-Aid Mix (Kraft Foods) in the water to encourage consumption. The antibiotic solution was passed through a sterilizing, 0.22-μm, nonpyrogenic cellulose acetate filter (Corning) before delivery to mice and was replaced with freshly prepared solution two to three times per week. For analysis of weight gain on antibiotics, mice were tracked over a 3-wk treatment period from 24 to 45 d of age.

### Statistical Analysis

Statistical analysis was performed using Prism v4.03 (GraphPad Software) and Stata10 (StataCorp) software. To compare data obtained from the analysis of WT, dnTGFβRII, IL-10R2^−/−^, and dnKO mice, a one-way analysis of variance (*F* test) was used to determine if statistically significant differences existed between groups; the degrees of freedom for each *F* test were indicated in brackets (treatment, residual). In cases where the *F* test revealed such differences (*p* < 0.05), a Bonferroni's multiple comparison post-test was used to determine statistical significance between any two groups (*p* < 0.05 was considered statically significant). Similar analysis was employed for T cell transfer experiments and anticytokine treatments. The Kaplan-Meier method was used to analyze survival of T cell transfer recipient and antibiotic-treated mice. Statistical significance of survival differences between groups was calculated in GraphPad Prism using the log-rank test. To analyze and compare growth data, generalized estimating equations were generated using Stata10 software.

## Results

### Failure to Thrive Followed by Rapid Weight Loss and Early Mortality in dnKO Mice

Intestinal homeostasis is partially maintained through the actions of inhibitory cytokines that regulate mucosal inflammation. A single defect in either T cell TGFβRII or global IL-10R2 signaling results in spontaneous colitis induction. However, the ability to further study pathogenesis and treatments for IBD in these systems is limited because of the variability in penetrance of pathology and delayed onset of disease (typically up to 3–4 mo) observed in both models [8,9]. Human IBD shows a spectrum of disease severity that includes a population of patients who present with acute, fulminant, and life-threatening disease. The basis for this more severe form of disease is unclear, and we hypothesized that it may reflect a culmination of impairments to multiple genes. Therefore, we wanted to establish whether combining multiple genetic defects in immune regulation would be sufficient to significantly exacerbate colitis.

**Figure 1:**
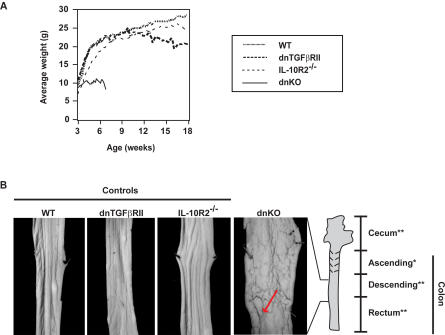
dnKO Mice Fail to Thrive and Waste Rapidly Because of a Fatal, 100% Penetrant Disease Process Localized to the Cecum and Colon (A) Plot of the average weight/group versus time for WT (*n* = 4), dnTGFβRII (*n* = 4), IL-10R2^−/−^ (*n* = 5), and dnKO (*n* = 11) mice. All mice were weighed two or three times per week from 3 to 18 wk of age. Error bars were omitted for the sake of clarity. In all cases the experimental error was ≤ 8% of the mean value. Individual mice died or were humanely killed when their weight reached ≤ 70% of their maximal weight. The line representing dnKO average weight terminates with the death of the final dnKO mouse. (B) Whole-mount images of the mucosal surface of the descending colon from 4- to 5-wk-old WT, dnTGFβRII, IL-10R2^−/−^, and dnKO mice. The cecum and entire colons were harvested, dissected, opened, pinned in Bouin's fixative, and examined for gross morphology. The dnKO mice all contained major pathologic alterations including severe ulceration (e.g., red arrow) and mucosal thickening in the cecum, descending colon, and rectum (**), as well as minor alterations (*) in the ascending colon.

We bred dnTGFβRII mice with IL-10R2^−/−^ mice to generate a novel mouse strain, the dnTGFβRII × IL-10R2^−/−^ (dnKO), which has impaired TGFβRII and IL-10R2 signaling in the T cell compartment and deficiencies in IL-10R2 dependent signaling in all cells. Total body weights of dnKO and controls (consisting of WT, dnTGFβRII, and IL-10R2^−/−^ mice) showed that dnKO mice failed to thrive by 3–4 wk and demonstrated rapid weight loss culminating in death as compared to all controls by 4–6 wk (Figure 1A). This phenotype was 100% penetrant in dnKO mice, and no apparent gender difference in weight loss was observed (unpublished data). These data indicate that the kinetics of disease induction was significantly accelerated through the combination of two separate genetic deficiencies.

### Development of Spontaneous Fulminant Ulcerative Colitis upon Loss of TGFβRII and IL-10R2 Signaling

Because individually both dnTGFβRII and IL-10R2^−/−^ mice spontaneously develop colitis, we wanted to establish whether the colon was the primary target and cause of the wasting phenotype detected in dnKO mice. Gross and histological surveys of dnKO mice ≥4 wk of age revealed only small, occasional clusters of lymphocytes located in the portal triads of the liver and around bronchi of the lungs, without any tissue destruction (unpublished data). No abnormalities were observed in the heart, kidney, stomach, and small intestine of dnKO mice.

However, at this time, striking disruptions in the gross morphology of the entire cecum, descending colon, and rectum in dnKO mice were observed, with the overall architecture of the ascending colon being relatively spared. The cecum (unpublished data) and descending colon/rectum areas (Figure 1B) of dnKO mice showed a diffuse and marked thickening of the mucosa accompanied by areas of focal ulceration (Figure 1B). This phenotype was severe, confluent, regionally consistent, and 100% penetrant in all dnKO mice.

Analysis of WT, dnTGFβRII, and IL-10R2^−/−^ control mice at 4–5 wk of age revealed that the cecum and colons were generally unaffected. We detected colonic pathology in only dnTGFβRII and IL-10R2^−/−^ mice that were considerably older (3–4 mo). In contrast to the diffuse mucosal thickening that is the hallmark of dnKO mice, dnTGFβRII and IL-10R2^−/−^ mice colons contained a focal and variable disease process that typically resulted in ulcerations found near junctions, including the ileal-cecal junction, ascending-to-descending colon transition and the anal-rectal junction (Figure S1).

**Figure 2:**
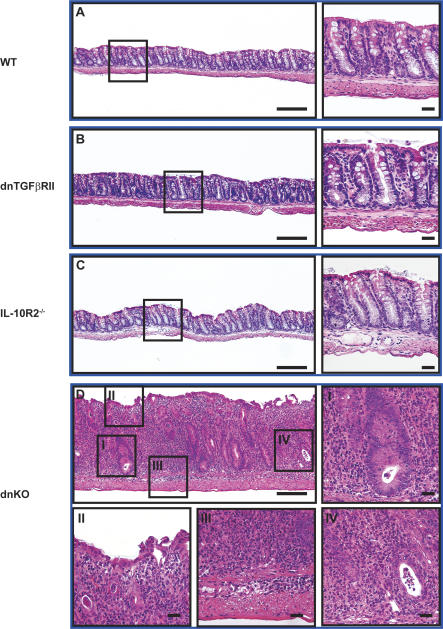
dnKO Mice Develop Diffuse Fulminant Ulcerative Colitis That Is Not Detected in Age-matched Controls Colons from WT, dnTGFβRII, IL-10R2^−/−^, and dnKO mice were isolated at 4–5 wk of age as in Figure 1. Images from HE stained sections of rectums from (A) WT, (B) dnTGFβRII, (C) IL-10R2^−/−^, and (D) dnKO mice are shown at low power (100×). One higher power image (400×) is shown for (A–C, right images) and four higher-power images (I–IV) for (D). The boxed region on the lower power image indicates the location of the higher magnification(s). The four highlighted regions in the dnKO image (D) reflect (I) epithelial hyperplasia, presence of increased M-phase cells, and goblet cell loss in crypts; (II) eroded surface epithelium; (III) mucosal and submucosal leukocytic inflammation; and (IV) the presence of a crypt abscess. Bars, 200 μm for 100× images and 30 μm for 400× images.

Histological examination of WT, dnTGFβRII, and IL-10R2^−/−^ colons revealed relatively normal intestinal architecture at 4–5 wk of age (Figure 2A–2C). In contrast, dnKO mice had severe and diffuse alterations in mucosal structure that correlated with the gross findings described above and included marked extensive epithelial hyperplasia, diminished goblet cell and crypt number, erosion of surface epithelial cells, numerous crypt abscesses, and mixed leukocytic infiltrates localized in both the mucosa and submucosa of the cecum, descending colon, and rectum (Figure 2D and unpublished data). Although leukocytic infiltrates were detected in the ascending colon, the epithelium and goblets cells in this region were relatively spared (unpublished data).

**Figure 3:**
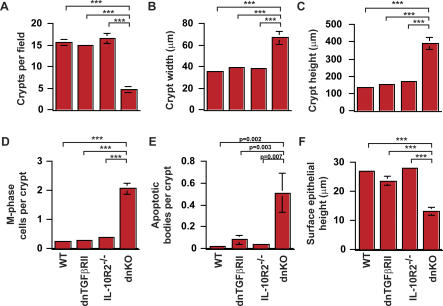
dnKO Histopathology Shares Features of Inflammation Detected in Human Ulcerative Colitis Objective, quantitative morphometric analysis of the rectal histopathology was conducted on 4–5-wk-old WT, dnTGFβRII, IL-10R2^−/−^, and dnKO mice. (A–D) Using well-oriented sections, we measured crypt loss/drop out by examining (A) the number of crypts per field (a field, 870 μm) and (B) crypt width. Epithelial hyperplasia was assayed by measuring (C) crypt height (from the base of the crypt to the basal side of the surface epithelial cells) and (D) the ratio of M-phase cells/crypt. (E) The ratio of apoptotic bodies/crypt and (F) surface epithelial heights were quantified to determine effects on cell death and barrier epithelial changes. All measurements are the averages ± SEM from *n* = 5–8 mice per group. The *F* test results are (A) *F*(3,23) = 26.74, *p* < 0.0001; (B) *F*(3,23) = 21.17, *p* < 0.0001; (C) *F*(3,23) = 53.53, *p* < 0.0001; (D) *F*(3,25) = 89.47, *p* < 0.0001; (E) *F*(3,24) = 7.574, *p* = 0.001; (F) *F*(3,23) = 41.35, *p* < 0.0001. Where *F* testing revealed statistically significant differences within groups (*p* < 0.05), individual means were compared by Bonferroni's multiple comparison test. All statistically significant comparisons (*p* < 0.05) between any two groups were indicated with a bracket. The exact *p*-value is listed unless *p* < 0.001 (indicated by ***).

Because the pathology of dnKO mice was diffuse and consistent, we were able to objectively quantify specific features of the histopathology that reflect disease severity instead of utilizing histology scores that are generally more effective for focal and variable disease processes. For example, in the rectums of dnKO mice, we found a statistically significant loss of crypts, as demonstrated by decreased numbers of crypts per field (each field, 870 μm) and increased crypt width as compared to all controls (Figure 3A and 3B). Additionally, dnKO mice showed significant epithelial hyperplasia indicated by increased crypt height (Figure 3C) and elevated M-phase bodies per crypt ratios (Figure 3D), a response that is typical of injury in this organ [22]. We also noted an increased number of apoptotic bodies per crypt (Figure 3E) and decreased surface epithelial cell heights (Figure 3F) in dnKO mice. All of these parameters are indicative of a mucosal response to inflammatory stimulus (e.g., [22]).

**Figure 4:**
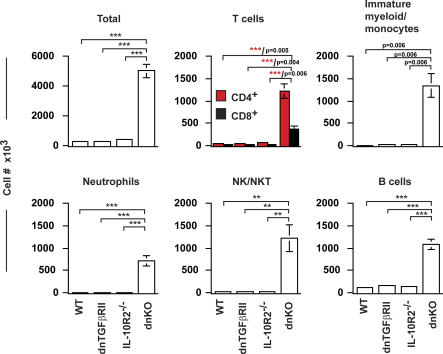
Diseased dnKO Mice Have Diverse Leukocytic Infiltrates Located in the Cecum and Colon Lamina propria/MALT cells from the pooled cecum, descending colon, and rectum were isolated from 4–5-wk-old WT, dnTGFβRII, IL-10R2^−/−^, and dnKO mice. Shown are the averages ±SEM for the total number of CD45.2^+^ (hematopoietic) cells located within the tissue as well as for each subset of CD45.2^+^ cells stained for T cells (CD4^+^ or CD8^+^), immature myeloid/monocytes (CD11b^+^Gr1^lo^), neutrophils (CD11b^+^Gr1^hi^), NK/NKT (DX5^+^), and B cells (B220^+^) from seven separate experiments with *n* = 6–15 mice per group. Results were consistent with observations using immunofluorescence microscopy (not shown). The *F* test results are: total immune cells, *F*(3,32) = 69.76, *p* < 0.0001; CD4^+^ T cells, *F*(3,32) = 23.16, *p* < 0.0001; CD8^+^ T cells, *F*(3,32) = 9.779, *p* < 0.0001; monocytes/immature myeloid cells, *F*(3,16) = 11.57, *p* = 0.0003; neutrophils, *F*(3,16) = 16.59, *p* < 0.0001; NK/NKT cells, *F*(3,16) = 10.93, *p* = 0.0005; B cells, *F*(3,27) = 27.47, *p* < 0.0001. All statistically significant comparisons (*p* < 0.05) between any two groups are indicated with a bracket. The exact *p*-value is listed unless *p* < 0.001 (indicated by ***).

In accordance with the histological data reflecting intestinal inflammation, isolation of lamina propria and MALT infiltrates in the cecum, descending colon, and rectal regions of dnKO and control mice demonstrated a 10-fold increase in cell numbers in the dnKO mice (Figure 4). The cellular composition consisted mainly of T cells, immature myeloid/monocytes (CD11b^+^Gr1^lo^), neutrophils (CD11b^+^Gr1^hi^), NK/NKT (DX5^+^), and B cells (B220^+^) (Figure 4). The increase in the cell number by this method correlates with that seen in tissue sections from control and dnKO mice stained with antibodies for markers of each immune cell type (unpublished data).

Taken together, the findings of a severe inflammatory process in the dnKO mouse colon, which show (i) localization to the cecum and colon (the distal small intestine is not involved; Figures 1B, S1B and unpublished data); (ii) primarily mucosal damage (the muscularis is relatively spared; Figure 2D); (iii) diffuse distribution (no skip areas; Figures 1B and S1B); (iv) numerous crypt abscesses (Figure 2D); (v) marked mucosal regenerative response (Figures 2D and 3D); (vi) loss of goblet cell mucin (Figure 2D); (vii) focal punctate ulcers (no longitudinal ulcers or fissures; Figures 1B and S1B); (viii) lack of prominence of granulomas (Figure 1B); and (ix) no colonic fistulas or strictures, all indicate that the disease process is most representative of ulcerative colitis and not Crohn's disease [23].

### Elevated Circulating Proinflammatory Cytokines and Early in Vivo Activation of T Cells in dnKO Mice

**Figure 5:**
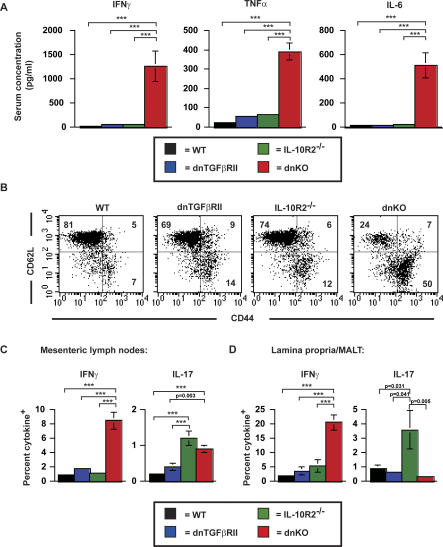
Colitis in dnKO Mice Is Associated with Elevated Proinflammatory Cytokine Levels and Increased T Cell Activation (A) Plot of average serum concentrations of IFNγ, TNFα, and IL-6 of 4–5-wk-old WT, dnTGFβRII, IL-10R2^−/−^, and dnKO mice. Shown are the average ± SEM from *n* = 10–12 mice/group. The *F* test results are: INFγ, *F*(3,38) = 11.73, *p* < 0.0001; TNFα, *F*(3,38) = 48.32, *p* < 0.0001; IL-6, *F*(3,38) = 18.41, *p* < 0.0001. Brackets denote all statistically significant differences between two groups; ***p ≤ 0.0001 as described in Materials and Methods. (B) A representative example of CD62L versus CD44 profile of CD4^+^ T cells from the mesenteric (draining) lymph nodes depicting decreased percentages of naive (CD62L^hi^CD44^lo^) cells accompanied by increased percentages of effector/memory cells (CD62L^lo^CD44^hi^) in dnKO mice compared to controls. Similar results were observed in five to seven separate experiments from *n* = 6–12 mice/group. (C and D) The percentages of CD4^+^ IFNγ^+^ or IL-17^+^ cells were determined in the (C) mesenteric (draining) lymph nodes and (D) lamina propria/MALT of 4–5-wk-old mice WT, dnTGFβRII, IL-10R2^−/−^, and dnKO mice. Shown is the average ± SEM from *n* = 6–11 mice per group. The *F* test results are: mesenteric lymph node INFγ, *F*(3,33) = 37.52, *p* < 0.0001; mesenteric lymph node IL-17, *F*(3,33) = 21.16, *p* < 0.0001; lamina propria INFγ, *F*(3,23) = 20.34, *p* < 0.0001; lamina propria IL-17, *F*(3,23) = 7.119, *p* < 0.0001. All statistically significant comparisons (*p* < 0.05) between any two groups are indicated with a bracket. The exact *p*-value is listed unless *p* < 0.001 (indicated by ***).

Human IBD has been associated with elevated proinflammatory cytokines, including IFNγ and TNFα [24–26], and regulation of these types of cytokines is often mediated via signals received through TGFβRII and IL-10R2. Therefore, we wanted to determine whether proinflammatory cytokines were significantly altered in dnKO mice during disease. Analysis of circulating IFNγ, TNFα, and IL-6 levels in the serum obtained from 4- to 5-wk-old dnKO mice revealed a striking increase in the concentrations of all three cytokines in comparison to control mice (Figure 5A). Serum from dnKO mice had mean levels of 1,268.5 pg/ml of IFNγ, 390.9 pg/ml of TNFα, and 516.1 pg/ml of IL-6, which respectively represented at least an 18-fold, 6-fold, and 19-fold induction over the highest levels seen in controls.

We hypothesized that T cells might be responsible for the observed increases in proinflammatory cytokine production. To assess whether the combined loss of both IL-10R2 and TGFβRII signaling resulted in a more dramatic alteration in activation status, the phenotypes of WT, dnTGFβRII, IL-10R2^−/−^, and dnKO T cells were examined. At 4–5 wk of age, decreases in the percentages of CD62L^hi^ CD44^lo^ naive cells were accompanied by increased percentages of CD62L^lo^ CD44^hi^ effector/memory cells in both CD4^+^ and CD8^+^ T cell compartments of the mesenteric lymph nodes of dnKO mice as compared to all controls (Figure 5B and unpublished data). Increases in activated and effector/memory cells in dnTGFβRII mice as compared to WT controls were also detected, consistent with a previous study by Gorelik et al. [8]. At 3 wk, overtly healthy dnKO mice had similar levels of naive, activated, and effector/memory CD4^+^ and CD8^+^ T cells as compared to controls (unpublished data). T cell development in dnKO mice also appeared grossly normal prior to disease as similar CD4 and CD8 profiles were seen in the thymus of overtly healthy dnKO mice. In contrast, disease in dnKO mice was accompanied by thymic involution because of the loss of CD4^+^CD8^+^ thymocytes (Figure S2). Additionally, similar percentages of CD4^+^CD25^+^Foxp3^+^ regulatory T cells were detected in the draining lymph node and in the intestines of dnKO mice as compared to controls (Figure S3). Thymic regulatory T cells from these mice also displayed intact inhibitory function in vitro (Figure S3). Together, these data suggest that the T cell compartment in dnKO mice was initially normal, but became rapidly activated during disease.

Next we determined if T cell effector function was altered in dnKO mice by examination of cytokine production. At ≥4 wk of age, dnKO mice had significantly higher percentages (Figure 5C) of IFNγ^+^ CD4^+^ T cells over all controls and enhanced percentages of IL-17^+^ CD4^+^ T cells compared to WT and dnTGFβRII, but not IL-10R2^−/−^ mice, in the mesenteric lymph nodes. Additionally, the percentage of TNFα^+^ CD4^+^ cells was elevated in both dnTGFβRII (32.6 ± 2.4%) and dnKO (36.2 ± 3.4%) mice as compared to WT (23.8% ± 2.5%) and IL-10R2^−/−^ (22.4% ± 4.1%) controls. Increases in IFNγ^+^ CD8^+^ T cells were also seen in dnTGFβRII, IL-10R2^−/−^, and dnKO mice in comparison to WT mice (unpublished data). Isolation of T cells from the lamina propria/MALT of the cecum and colon revealed a significantly elevated percentage of IFNγ^+^ CD4^+^ T cells in dnKO mice at the site of inflammation. In contrast, the percentages of TNFα^+^ and IL-17^+^ CD4^+^ T cells in the colons of dnKO mice were similar or lower than controls (Figure 5D and unpublished data). Elevated levels of IFNγ and TNFα were also detected in colon explants from dnKO mice as compared to controls (unpublished data). Because TNFα was increased in both the serum and colon explants but the percentages of TNFα^+^ T cells were similar to dnTGFβRII mice, this suggests that other cells (e.g., macrophages) are generating more of this particular cytokine. Together these data showed that multiple genetic hits to immune regulation signaling pathways resulted in significant exacerbation of T cell activation and effector function.

### dnKO CD4^+^ T Cells Transfer Disease in RAG^−/−^ Mice

**Figure 6:**
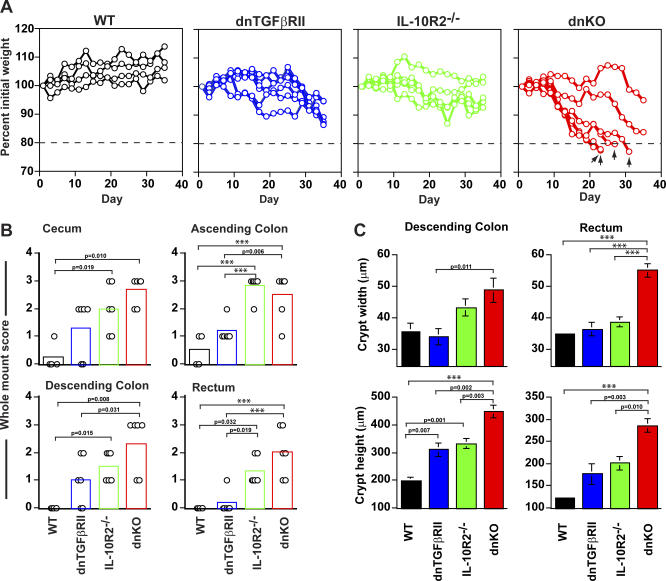
Transferred CD4^+^ T Cells from dnKO Mice Induce Fulminant Ulcerative Colitis in RAG1^−/−^ Mice CD4^+^ T cells were purified from ∼3-wk-old WT, dnTGFβRII, IL-10R2^−/−^, and dnKO mice and 2 × 10^6^ cells were transferred IP into B6.RAG1^−/−^ hosts. (A) Weights were recorded for mice that had received CD4^+^ T cells from WT (black, *n* = 4), dnTGFβRII (blue, *n* = 6), IL-10R2^−/−^ (green, *n* = 6), or dnKO (red, *n* = 6) mice. Shown for each group is the percent initial weight of individual mice from three separate experiments. The upward arrow indicates individual mice that were humanely killed for analysis when their weight reached ≤ 80% of their maximal weight (dashed lines). Analysis using the Kaplan-Meier method showed the difference between groups of mice reaching this endpoint of weight loss was statistically significant (*p* < 0.001, df = 3, log-rank test). (B) Whole mount photographs of the cecum, ascending colon, descending colon, and rectum were scored in a blinded fashion on a 0–3 scale. 0, normal; 1, focal ulcers present; 2, ulcers and diffuse, mild mucosal thickening; and 3, ulcers and diffuse, severe mucosal thickening. The bars represent the average whole-mount score, with each circle representing an individual mouse described in (A). The *F* test results are: cecum, *F*(3,18) = 8.07, *p* = 0.0013; ascending colon, *F*(3,18) = 17.93, *p* < 0.0001; descending colon, *F*(3,18) = 7.720, *p* = 0.0016; rectum, *F*(3,18) = 14.39, *p* < 0.0001. All statistically significant differences (using Bonferroni's multiple comparison test; *p* < 0.05) between any two groups were indicated with a bracket. The exact *p*-value is listed unless *p* < 0.001 (indicated by ***). Scores for recipients receiving dnKO CD4^+^ T cells were significantly higher than those receiving either WT or dnTGFβRII CD4^+^ T cells in the ascending colon, descending colon, and rectum. (C) The height and width of the crypts in the descending colon and rectum of HE stained tissue samples were analyzed to measure epithelial hyperplasia and crypt drop out of the mice described in (A). The bar graphs represent the average crypt height or width ± SEM in the descending colon or rectum from B6RAG1^−/−^ mice that received CD4^+^ T cells from the indicated donors. The *F* test results are: DC crypt width, *F*(3,18) = 5.061, *p* = 0.0102; DC crypt height, *F*(3,18) = 20.23, *p* < 0.0001; rectum crypt width, *F*(3,18) = 13.82, *p* < 0.0001; rectum crypt height, *F*(3,18) = 23.58, *p* < 0.0001. All statistically significant differences (using Bonferroni's multiple comparison test; *p* < 0.05) between any two groups are indicated with a bracket. The exact *p*-value is listed unless *p* < 0.001 (indicated by ***). Mice that received dnKO CD4^+^ T cells had statistically significant increases in crypt height in the descending colon and rectum compared to all controls and wider crypts in the rectum compared to all controls. Significant increases in the width of descending colon crypts in recipients receiving dnKO CD4^+^ T cells as compared to recipients receiving WT or dnTGFβRII were also observed.

To examine directly the role of T cells in the induction of ulcerative colitis in the dnKO mice, we utilized the established model of CD4^+^ T cell transfer into immunodeficient recipients [27]. CD4^+^ T cells were isolated from WT, dnTGFβRII, IL-10R2^−/−^, and dnKO mice and transferred to B6.RAG1^−/−^ mice. Recipient mice were humanely killed and their colons analyzed at either 5 wk post-transfer or upon loss of >20% of their initial body weight, whichever occurred first. Weight loss of >20% within 5 wk of transfer was observed in four of six mice in the dnKO T cell recipient group but none of the animals in the other groups (*p* < 0.001; Figure 6A).

Gross and histologic examination of colons from recipient mice showed a trend whereby transfer of T cells from dnKO donors produced more severe colitis as compared to transfer of T cells from either dnTGFβRII, IL-10R2^−/−^ or WT donors (Figure 6B and 6C). Transfer of dnKO CD4^+^ T cells induced a significant increase in the crypt height and width in the rectum as well as crypt height in the descending colon as compared to mice that received CD4^+^ T cells from the WT, dnTGFβRII, or IL-10R2^−/−^ control groups (Figure 6C). Because crypt height and width reflected the amount of hyperplasia and crypt dropout that had occurred within the colon, these measurements were indicative of severe colitis induced by the dnKO CD4^+^ T cells. These studies demonstrate that CD4^+^ T cells were directly involved in the induction of colitis in the dnKO mice.

### Neutralization of IFNγ and TNFα Significantly Ameliorates Disease in dnKO Mice

**Figure 7:**
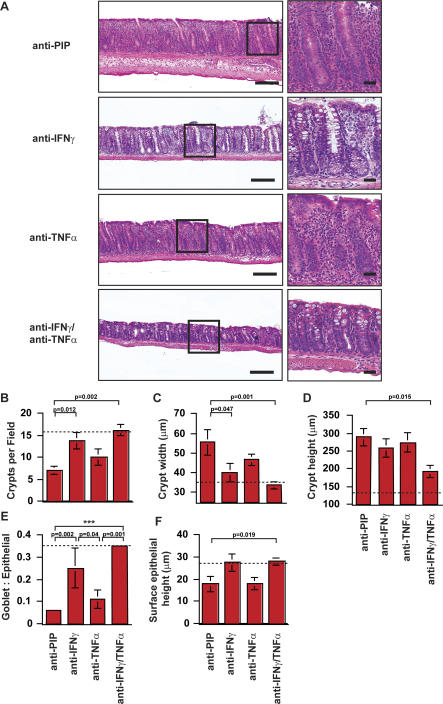
Amelioration of Colitis in dnKO Mice Through Simultaneous Neutralization of IFNγ and TNFα (A) HE-stained sections of the rectums of dnKO mice treated with neutralizing antibodies to inflammatory cytokines. Colons from dnKO mice injected IP with 1 mg of neutralizing antibodies against IFNγ, TNFα, IFNγ + TNFα, or an isotype control (anti-PIP) at 2 and 3 wk of age were harvested at 4 wk of age. (B–F) Quantitative morphometric analysis of the histopathology from dnKO mice treated with anti-PIP (isotype control), anti-IFNγ, anti-TNFα, or a combination of anti-IFNγ and anti-TNFα is shown. Crypt loss/dropout was measured by examining (B) the number of crypts per field (a field, 870 μm) and (C) crypt width. Epithelial proliferation was measured by (D) crypt height taken from the base of the crypt to the basal side of the surface epithelial cells. (E and F) The ratio of goblet cells/epithelial cells per crypt (E) and surface epithelial heights (F) were quantified to examine goblet cell loss and barrier changes. Shown for all measurements are the averages ± SEM from *n* = 5 mice per group. The *F* test results are (B) *F*(3,17) = 7.627, *p* = 0.0019; (C) *F*(3,17) = 7.467, *p* = 0.0021; (D) *F*(3,17) = 4.326, *p* = 0.0194; (E) *F*(3,17) = 11.45, *p* = 0.0002; and (F) *F*(3,17) = 3.664, *p* = 0.0334. All statistically significant differences (using Bonferroni's multiple comparison test; *p* < 0.05) between any two groups were indicated with a bracket. The exact *p*-value is listed unless *p* < 0.001 (indicated by ***).

Since IFNγ and TNFα are present at elevated levels in dnKO mice during disease, we neutralized these cytokines either singly or in combination to determine if doing so would limit disease. Well-characterized antibodies against IFNγ, TNFα, or both were administered at 2 and 3 wk of age in dnKO mice prior to disease assessment at 4 wk. Anti-IFNγ treatment alone significantly diminished loss of crypts, goblet cells, and surface epithelial cell height, but did not affect epithelial hyperplasia as compared to isotype control (Figure 7). In contrast, mice given anti-TNFα treatment alone did not have any significant alterations in colitis induction. Interestingly, neutralization of both IFNγ and TNFα resulted in even greater amelioration of pathology in dnKO mice than single therapy alone, including significantly decreased epithelial hyperplasia. Therefore, colitis in dnKO mice is partly mediated by excessive IFNγ and TNFα production, and combination therapy to neutralize these cytokines significantly diminished most aspects of pathology.

### Treatment with Broad-Spectrum Antibiotics Completely Inhibits Disease in dnKO Mice

**Figure 8:**
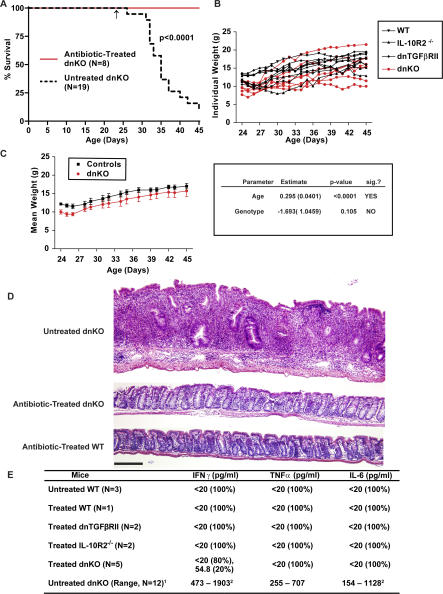
Inhibition of Colitis in dnKO Mice by Broad-spectrum Antibiotic Treatment (A) 45-d survival of untreated dnKO mice (*n* = 19; 45-d survival = 10.5%; median survival = 35 d) and dnKO mice receiving metronidazole and ciprofloxacin in drinking water (*n* = 8; 45-d survival = 100%). Individual mice died or were humanely killed when their weight reached ≤ 70% of maximal weight. Survival was analyzed by the Kaplan-Meier method, and statistical significance of difference between groups is *p* < 0.0001, by log-rank test. Upward arrow, antibiotic treatment begun at age 24 d. (B and C) Weight gain of antibiotic-treated mice (*n* = 2 WT, *n* = 3 IL-10R^−/−^, *n* = 4 dnTGFβRII, and *n* = 7 dnKO) plotted individually (B) or as mean weights (C) of treated dnKO mice (*n* = 7) and treated controls (*n* = 9; WT, IL-10R^−/−^, and dnTGFβRII combined) ± SEM. Data were pooled from three separate experiments using dnKO and littermate controls. To account for the longitudinal nature of the data, analysis of weight change over the course of treatment was performed using generalized estimating equations. The mice gained weight over the course of the experiment (*p* < 0.001), and the dnKO and control groups did not differ significantly (*p* = 0.105). (D) Representative images of HE stained sections of rectums from an untreated dnKO mouse humanely killed at ≥ 4 wk, an antibiotic-treated dnKO mouse, and an antibiotic-treated WT mice killed at 45 d of age are shown at low power (bars in [C–E], 200 μm). (E) Serum concentrations of IFNγ, TNFα, and IL-6 in individual untreated WT (*n* = 3) mice and antibiotic-treated WT (*n* = 1), dnTGFβRII (*n* = 2), IL-10R^−/−^ (*n* = 2), and dnKO (*n* = 5) mice measured at 5–6 wk. All concentrations were below the limit of detection (<20 pg/ml) except for low levels of IFNγ in one antibiotic-treated dnKO mouse (54.8 pg/ml). ^1^Ranges of cytokine concentrations from the experiment depicted in Figure 5A are shown for comparison. ^2^Indicates exclusion of outlier values: one mouse had 4,493 pg/ml IFNγ, and one had 75 pg/ml IL-6.

Studies in a variety of murine models have shown an important role for intestinal bacteria in inducing colitic disease [28]. At 2 wk of age, the intestines of dnKO mice appeared normal both macroscopically and microscopically (unpublished data). This observation indicated that general intestinal architecture development in these mice was not perturbed at this time. Disease induction was rapid and could be detected by 3 wk of age, a time commensurate with known changes in the microbial ecology of the mammalian intestine that occur at the weaning–suckling transition [29]. To examine whether bacteria influence disease development in the dnKO model, we treated mice with broad-spectrum antibiotics in drinking water. Beginning at 24 d of age, mice received drinking water containing high-dose ciprofloxacin and metronidazole, two commonly used antibiotics that are broadly active against aerobic and anaerobic bacterial species, respectively [30]. In striking contrast to untreated animals, antibiotic-treated dnKO mice exhibited 100% 45-d survival (Figure 8A; *p* < 0.0001). We were thus able to compare the growth of treated dnKO mice with treated littermate controls (WT, dnTGFβRII, and IL-10R2^−/−^ combined) over a 3-wk treatment period (Figure 8B). Both groups of mice showed weight gain over the course of the experiment. No statistically significant difference in weight gain was observed between the dnKO and control groups (*p* = 0.105; Figure 8C).

Consistent with survival and growth observations, gross and histologic examination of intestines from all antibiotic-treated dnKO mice aged 45–48 d revealed no inflammation, mucosal thickening, or ulceration throughout the cecum and colon, with dnKO intestines appearing essentially identical to controls (Figure 8D and unpublished data). The quantitative morphometric measures of inflammation described previously did not differ significantly between treated dnKO mice and controls (unpublished data).

In accordance with the absence of inflammation and mucosal barrier damage, analysis of serum from antibiotic-treated mice revealed near-complete abrogation of proinflammatory cytokine production. In contrast to untreated dnKO mice (Figure 5A), mice on antibiotics had undetectable levels of TNFα and IL-6, and IFNγ was detectable at low levels in only one of five animals assayed (Figure 8E). Antibiotics also decreased cytokine production in dnTGFβRII and IL-10R2^−/−^ mice to undetectable levels (Figures 5A and 8E). Collectively, these results demonstrate that broad-spectrum antibiotic treatment completely inhibits ulcerative colitis in dnKO mice.

## Discussion

In this report, we demonstrate multiple genetic deficiencies in immune regulation result in fulminant ulcerative colitis that is distinct from and more severe than the disease induced by either of the single deficiencies alone. In addition to documenting these pathologic findings, we further show that disease in dnKO mice is characterized by elevated proinflammatory cytokines, can be induced in recipient animals by transfer of dnKO CD4^+^ T cells, is responsive to combined neutralization of IFNγ and TNFα, and can be completely inhibited through treatment with high-dose oral ciprofloxacin and metronidazole.

Previous work has shown that the inhibitory cytokines IL-10 and TGFβ, both known to control immune homeostasis, are associated with protection from colitis in murine models of IBD [7–9,31–35]. We crossed dnTGFβRII mice, which have significantly attenuated TGFβRII signaling in T cells, with IL-10R2^−/−^ mice. The resulting dnKO mice showed a highly reproducible, diffuse, and severe ulcerative colitis that occurred in a predictable time frame. At the same time point at which dnKO mice developed severe disease and died, all controls including WT, dnTGFβRII, and IL-10R2^−/−^ mice demonstrated little to no pathology. Similarly, work in IL-10^−/−^ mice has demonstrated differential colitis severity in a C3H/HeJBir versus a C57BL/6J background and linked the differences to a region on Chromosome 3, referred to as the cytokine deficiency-induced colitis susceptibility 1 (Cdcs1) [10,11]. These findings all support the idea that IBD is a complex disease influenced by multiple interacting host genetic factors.

Because both dnTGFβRII and IL-10R2 signaling are important for various aspects of immune regulation, the disease observed in dnKO mice was likely due to a culmination of several dysregulated parameters. The recent generation of T cell specific TGFβRII knockout mice revealed that this defect alone was sufficient to induce rapid, multiorgan autoimmunity. These mice demonstrated alterations in Th1 T cell differentiation, NKT cell development, and impaired maintenance, but not generation, of Foxp3 regulatory T cells [36,37]. Similarly, in dnKO mice, regulatory T cells were present at similar percentages to controls, suggesting that regulatory T cell development was also intact in these mice. Furthermore, dnKO T regulatory cells were able to inhibit naive T cell proliferation in vitro, suggesting that they still are able to function in this context. Regulation of a T cell transfer model of colitis has previously been demonstrated to depend on TGFβ responsiveness by pathogenic naive T cells [31], emphasizing the importance of this cytokine in disease inhibition. We showed that CD4^+^ T cells from dnTGFβRII mice could also transfer disease, but found that the additional loss of IL-10R2 signaling increased the severity of disease above that conferred by cells in which only TGFβRII signaling was disrupted. These studies, however, did not address whether the loss of IL-10R2 and TGFβRII signaling caused dysregulation of the naive or regulatory T cell subsets found within the CD4^+^ population [38].

In addition to impaired TGFβRII signaling, IL-10R2 signaling was completely absent in dnKO mice. As a result, these mice were unresponsive to IL-10, which normally inhibits antigen-presenting cell function and may play a role in converting naive CD4^+^ T cells to those with regulatory potential [12,14,39–42]. The importance of IL-10 in regulation of colitis was initially demonstrated in IL-10^−/−^ mice that developed spontaneous enterocolitis marked by crypt abnormalities, epithelial hyperplasia, and leukocytic infiltrates [7]. The generation of IL-10R2^−/−^ mice recapitulated data from IL-10^−/−^ mice in that they also spontaneously developed colitis marked by epithelial hyperplasia and leukocytic infiltrates. Interestingly, IL-10 has also been implicated in contributing to epithelial barrier integrity [43], which would be a critical aspect in limiting intestinal mucosal inflammation.

Because the IL-10R2 is also a redundant receptor for other cytokines, including IL-22, IL-26, IL-28, and IL-29 [44], the effects of these cytokines on colitis development in dnKO mice could not be ruled out. While little is known about the relevance of most of these cytokines in the context of intestinal immunity, increased levels of IL-22 have been noted in human IBD patients, and IL-22 has also been demonstrated to induce IL-10 expression by colon epithelial cell lines [45,46]. However, abrogation of signaling in these cells may be beneficial, as IL-22 has also been shown to induce production of proinflammatory cytokines by colonic subepithelial myofibroblasts and intestinal epithelial cells [45,47]. Additionally, previous characterization of IL-10R2^−/−^ mice did not reveal an increased disease severity compared to what was described for IL-10^−/−^ mice [7,9]. With the given caveat that there may have been differences in enteric flora, the data from these studies suggest that IL-10, but not the other redundant IL-10R2 cytokines, plays the predominant role in colitis induction and exacerbation.

In addition to potential alterations in APC function, epithelial barrier integrity, and T regulatory-mediated suppression, a major function attributed to both IL-10 and TGFβ is the inhibition of proinflammatory cytokine production [12–16]. Excessive production of proinflammatory cytokines has been implicated as a potential factor in human IBD [24–26]. For example, CD4^+^ lamina propria cells from patients with Crohn's disease produced increased levels of IFNγ and TNFα [24]. Furthermore, neutralization of TNFα via administration of infliximab resulted in clinical benefits for IBD patients and a variety of other such biologic agents are in various stages of development and clinical testing [48]. Studies in murine colitis models have also shown reduced pathology upon neutralization of IFNγ or TNFα [49–52]. In dnKO mice, impairment or loss of both TGFβRII and IL-10R2 signaling resulted in intestinal pathology that was associated with dramatic increases in both T cell activation and IFNγ, TNFα, and IL-6 levels. Depletion of IFNγ and TNFα decreased the extent of pathology seen in 4-wk-old dnKO mice as compared to controls. While anti-IFNγ treatment alone partially ameliorated disease, anti-TNFα treatment alone had little to no impact on intestinal pathology. Interestingly, significant reductions in epithelial hyperplasia were only detected when the two treatments were combined, suggesting that in the dnKO model these two cytokines have redundant roles in inducing hyperplasia. Our data therefore suggest that treatment regimens that effectively neutralize multiple proinflammatory cytokines could result in more successful clinical responses.

In recent years the role of IL-12 versus IL-23 in chronic inflammation has been reevaluated upon the realization that both cytokines share the IL-12p40 subunit [53–55]. In both human trials and murine models of colitis, neutralization of the IL-12p40 subunit resulted in decreased pathology [56,57]. Recently, a mutation in the IL-23R has been negatively linked with human IBD, suggesting that reduced signaling through this receptor protects against disease [58]. Additionally, a study by Yen et al. has indicated a role for IL-23, rather than IL-12, in the induction of colitis due to its ability to promote IL-17 production [59]. However, the proinflammatory effects of IL-23 are not necessarily restricted to its effects on IL-17 producing cells as IL-23 can increase proliferation and IFNγ production by effector/memory T cells [55] and has been demonstrated to also increase Th1 responses in colitis models [60,61]. Because IL-17 secretion was detected in the supernatants of colon explants (unpublished data) from dnKO mice, albeit at lower levels compared to IFNγ and TNFα production, IL-17 and/or IL-23 may account for the residual pathology observed in dnKO mice after neutralization of both IFNγ and TNFα. Regardless, results from our study and others would suggest that large perturbations in immune homeostasis through production of excessive Th1, Th17, or Th2 cytokines all have the ability to cause intestinal pathology.

Several overlapping lines of evidence, primarily from murine models, point to involvement of enteric bacteria in inducing IBD (reviewed in [28]). For example, intestinal pathology in a number of models including IL-10^−/−^ mice was diminished in germ-free settings [62]. Similarly, colonic inflammation due to DSS-induced injury has been shown to be reduced in germ-free and antibiotic-treated mice [22,63]. Experiments employing antibiotics to treat murine models of spontaneous colitis have demonstrated variable degrees of success depending on the animal model and the antibiotics used (e.g., [64,65]). We chose to employ two orally delivered antibiotics at high dosages: ciprofloxacin, which broadly targets aerobic bacteria, and metronidazole, which is active against anaerobic organisms. Antibiotic treatment resulted in complete inhibition of disease in dnKO mice as demonstrated by survival, weight gain equivalent to controls, absence of macroscopic and microscopic signs of colitis, and near-complete abrogation of proinflammatory cytokine production. These findings dramatically demonstrate that intestinal microbes are required for disease induction in this model of ulcerative colitis.

As discussed previously, current efforts at developing treatments for various forms of IBD, particularly ulcerative colitis, have focused extensively on devising costly new biologic agents, many of which act by specifically targeting cytokines including TNFα and IFNγ [48]. Our results demonstrate that neutralization of these cytokines, particularly in combination, also produced significant amelioration of colitis in dnKO mice. Although antibiotics, including ciprofloxacin and metronidazole, are sometimes used to treat patients with IBD, research regarding their efficacy remains inadequate. Current clinical consensus holds that they are useful in managing septic complications of IBD and certain aspects of Crohn's disease. However, their utility in ulcerative colitis has not been adequately assessed [30,66,67], and recommendations vary regarding usage in patients with fulminant ulcerative colitis [48,68,69]. We know of no large, blinded, controlled clinical studies examining combined metronidazole and ciprofloxacin treatment in ulcerative colitis. Our results suggest that carefully controlled studies examining the benefits of combined ciprofloxacin and metronidazole in ulcerative colitis may be appropriate.

Overall, our studies support the hypothesis that multiple genetic hits to immune regulation have the ability to produce the most severe forms of IBD. While impairment of either TGFβRII or IL-10R2 signaling alone was sufficient to cause some aspects of disease, the extent of pathology was significantly increased once these deficiencies were compounded. Our data demonstrate that the mechanism was at least in part due to uncontrolled T cell activation resulting in increased proinflammatory cytokine production. We further show that the inciting stimulus for this immune activation is microbial in origin. We propose that this model will be useful in the future to evaluate the mechanism of loss/attenuation of specific mucosal barrier components (e.g., goblet cells, surface enterocyte maturation, and epithelial stem cells) that also occurs in human ulcerative colitis, to evaluate combinatorial therapies directed against the numerous proinflammatory cytokines that are elicited by disregulated leukocytes, and to elucidate the mechanisms by which enteric microbes trigger disease.

## Supporting Information

Figure S1 Intestinal Pathology in dnTGFβRII and IL-10R2^−/−^ Mice Develops More Slowly and Has a More Variable and Focal Pattern Than the Rapid, Diffuse Disease Seen in dnKO Mice Ceca and colons from dnTGFβRII and IL-10R2^−/−^ mice were isolated at 3–4 mo of age. (A) Mucosal whole-mount photomicrographs of the gross morphology of the descending colon/rectum region of two dnTGFβRII and IL-10R2^−/−^ mice are shown. Yellow dashed line outlines focal ulcerations.
(B) The cecum and entire colon of four individual 3- to 3.5-mo-old IL-10R2^−/−^ mice and two 4–5-wk-old dnKO mice are shown. Yellow dashed line outlines focal ulcerations; red dashed line outlines diffuse mucosal thickening; pink asterisk indicates ileal-cecal junction.
(8.2 MB TIF)

Figure S2 Thymic T Cell Development Is Not Obviously Perturbed in Overtly Healthy dnKO Mice (A) Representative CD4 versus CD8 thymic profiles of 3-wk-old WT, dnTGFβRII, IL-10R2^−/−^, and dnKO mice, which were overtly healthy, are shown. This is representative of three to four separate experiments with four to eight mice per group.
(B) Depletion of thymic CD4^+^CD8^+^ cells is shown in ≥4-wk-old diseased dnKO mice. The average ± standard error of the mean (SEM) of the thymic cellularity for ≥4-wk-old WT, dnTGFβRII, IL-10R2^−/−^, and dnKO is shown.
These results are generated from three to seven separate experiments, with *n* = 7–8 mice per group. The *F* test result for (B) is: *F*(3,26) = 43.14, *p* = 0.0001. Brackets denote statistically significant differences between the two groups; ***p ≤ 0.0001.
(591 KB TIF)

Figure S3 Regulatory T cells Are Present in dnKO Mice (A) Cells from mesenteric lymph nodes of WT, dnTGFβRII, IL-10R2^−/−^, and dnKO mice were isolated and stained for CD4, CD25, and Foxp3 expression. Shown are representative plots of CD25 versus Foxp3 staining, gated on CD4^+^ T cells.
(B) The percentages of CD4^+^ CD25^+^ Foxp3^+^ found in the lamina propria of the pooled cecum, descending colon, and rectum of 4-wk-old WT, dnTGFβRII, IL-10R2^−/−^, and dnKO mice is shown. Each bar represents the average ± SEM from three separate experiments.
(C) CD4^+^ CD8^−^ CD25^+^ regulatory T cells derived from thymi of 3-wk-old WT, dnTGFβRII, IL-10R2^−/−^, and dnKO mice, which were overtly healthy, were incubated with naive CD4^+^CD45RB^hi^ T cells at a 1:1 ratio and stimulated with irradiated antigen-presenting cells and anti-CD3.
Shown is the average ± SEM of the percent inhibition of proliferation induced by T regulatory cells derived from the indicated source. The results are generated from ≥3 separate experiments. The *F* test results are (B), *F*(3,16) = 2.104, *p* = 0.14 (ns); (C), *F*(3,14) = 2.15, *p* = 0.14 (ns).
(1.4 MB TIF)

## Abbreviations

dnKO: dnTGFβRII × IL-10R2^−/−^
dnTGFβRII: dnTGFβRII × IL-10R2^+/−^
HE: hematoxylin and eosin
IBD: inflammatory bowel disease
MALT: mucosally associated lymphoid tissue
WT: IL-10R2^+/−^
